# Surgery Is in Itself a Risk Factor for the Patient

**DOI:** 10.3390/ijerph19084761

**Published:** 2022-04-14

**Authors:** Verónica Aranaz-Ostáriz, María Teresa Gea-Velázquez De Castro, Francisco López-Rodríguez-Arias, Diego San José-Saras, Jorge Vicente-Guijarro, Alberto Pardo-Hernández, Jesús María Aranaz-Andrés, on behalf of the ESHMAD Director Group and External Advisers

**Affiliations:** 1 Department of General Surgery, Elche Universitary Hospital, C/Almazara 11, 03202 Elche, Spain; veronica.aranaz@gmail.com (V.A.-O.); franloarias@hotmail.com (F.L.-R.-A.); 2 Department of Preventive Medicine and Public Health, Sant Joan d’Alacant Hospital, Ctra, N-332, s/n, 03550 Sant Joan d’Alacant, Spain; teregea@gmail.com; 3 Department of Preventive Medicine and Public Health, Hospital Universitario Ramón y Cajal, IRYCIS, 28034 Madrid, Spain; jorge.vicente@salud.madrid.org (J.V.-G.); jesusmaria.aranaz@salud.madrid.org (J.M.A.-A.); 4 Department of Medicine and Medical Specialities, School of Medicine, IRYCIS, Alcalá University, 28034 Madrid, Spain; 5 General Subdirectorate for Healthcare Quality and Healthcare Cooperation, Ministry of Health of the Community of Madrid, 28013 Madrid, Spain; alberto.pardo@salud.madrid.org; 6 Department of Medicine and Medical Specialities, School of Medicine, Rey Juan Carlos University, 28933 Madrid, Spain; 7 CIBER of Epidemiology and Public Health (CIBERESP), 28029 Madrid, Spain

**Keywords:** adverse events, surgical intervention, medical errors, clinical safety, quality of care, patient safety

## Abstract

(1) Background: Adverse events (AE) affect about 1 in 10 hospitalised patients, and almost half are related to surgical care. The aim of this study is to determine the prevalence of AE in operated and non-operated patients in surgical departments in order to determine whether surgical treatment is a risk factor for AE. (2) Methods: A cross-sectional design that included 3123 patients of 34 public hospitals in the Community of Madrid determining the prevalence of AEs in operated and non-operated patients in surgical departments. (3) Results: The prevalence of AE in non-operated patients was 8.7% and in those operated was 15.8%. The frequency of AE was higher in emergency surgery (20.6% vs. 12.4%). The 48.3% of AEs led to an increase in hospital stay, and surgery was involved in 92.4% of cases. The most frequent AEs were related to hospital-acquired infection (42.63%), followed by those related to a procedure (37.72%). In the multivariate analysis, being operated on represented 2.3 times the risk of developing an AE. (4) Conclusions: Surgical sites are particularly vulnerable to AE. Surgical intervention alone is a risk factor for AE, and we must continue to work to improve the safety of both patient care and the working environment of surgical professionals.

## 1. Introduction

Chantler, in 1999, warned, “The practice of medicine in the past used to be simple, ineffective and relatively safe, and today it has become complex, effective, but potentially dangerous” [1]. Thus, from the beginning of the World Health Organization’s (WHO) concern for patient safety, surgery was considered one of the key issues [2,3].

Adverse events related to health care (AE) constitute a public health problem due to their frequency, impact, increasing trend, as well as their severity and, in many cases, their preventability. They affect about 1 in 10 hospitalised patients, and half of them are considered preventable [4,5,6,7,8].

Surgery is an essential component of health care, and due to the increasing incidence of oncological pathology, cardiovascular pathology, and trauma, its weight in health care systems is experiencing an upward trend. On the other hand, thanks to technological progress, which is growing exponentially year after year, pathologies that were not previously amenable to surgical treatment are now being operated on a daily basis. This, coupled with the increased life expectancy of patients, presents us with a scenario in which we are performing more complex surgeries on more complex patients. Every year, 234 million major surgical operations are performed worldwide, which means that 1 in every 25 people will undergo surgery each year throughout the world [9]. Of these 234 million, up to 25% will suffer postoperative complications, with a crude death rate after major surgery of 0.5–5%. On the other hand, almost half of the AEs suffered by hospitalised patients are related to surgical care, of which half are considered preventable [10,11].

There is consensus that surgical specialities are the areas where most AEs occur. Surgery has been estimated to be associated with the occurrence of AEs in 1.9–3.6% of all admissions, accounting for 46–65% of all AEs [12]. However, whether surgery constitutes a risk in itself has not been analysed.

The aim of this study is to determine the prevalence of AE in operated and non-operated patients in surgical departments in order to determine, in a pioneering manner, whether surgical treatment increases the risk for AE.

## 2. Materials and Methods

A descriptive observational study with a cross-sectional design that included 34 public hospitals in the Community of Madrid was applied [13,14].

All hospitalised patients were included. The sample was obtained by surveying all patients admitted to the hospitals at the time of the study (second week of May 2019). The AE had to be present on the day the observation was conducted and could have occurred during or before hospitalisation.

Subsequently, the sample was grouped and analysed according to the admission service or unit, obtaining two comparative samples: patients admitted to medical services and patients admitted to surgical services.

AE was defined as any incident related to health care that caused harm to the patient [4], as set out in the Conceptual Framework of the International Classification for Patient Safety published by the WHO [15]. An AE could be by healthcare-related infections, complications of a procedure, complications in nurse and auxiliary nurse care, adverse effects of medication, and another type that does not fulfil the previous criteria.

All hospitalised patients were screened using an adapted form used in previous studies [16,17]. Patients with any positive items were reassessed using the MRF2 questionnaire [18], which assessed the attribution of the AE to the harm presented by the patient, its preventability and impact, and opportunities for improvement during care.

Independent variables collected were: age, gender, admission type (unplanned or planned), length of stay, hospital complexity (tertiary, secondary, primary, support), intrinsic risk factors (renal failure, cardiovascular disease, neoplasia, chronic obstructive pulmonary disease (EPOC), immunodeficiency, neutropenia, liver cirrhosis, hypoalbuminemia, pressure ulcers, impaired mobility, sensory deficits, obesity, cardiovascular disease and active smoking), and extrinsic risk factors (ERF) (previous surgery, peripheral vascular catheter, central vascular catheter, urinary catheterisation, and intubation).

A descriptive analysis was carried out exploring the distribution of the main variables, using a bivariate analysis, Chi-square test, or Fisher’s exact test for categorical variables, and Student’s *t*-test or Mann–Whitney U-test for numerical variables according to normality criteria, as well as the analysis of variance. Finally, logistic regression models were developed to investigate the factors associated with the occurrence of AE, using independent variables related to hospitalisation, patient characteristics, and AE characteristics.

The study was approved by the Ethics Review Committee of the Hospital Ramón y Cajal (reference 057/19). The principles of anonymity and confidentiality of the information received were guaranteed in both data collection and analysis, both at patient level and in terms of participating professionals and centres.

## 3. Results

A total of 8307 patients admitted to medical and surgical services in the 34 public hospitals of the Community of Madrid were monitored, with an overall AE prevalence of 12.1%.

In this article, 3123 patients admitted to surgical services were analysed. Of these, 1989 patients underwent surgery, and 1134 patients were treated without surgery. We identified 315 patients with AE in the operated group and 99 in the non-operated group.

**Figure 1:**
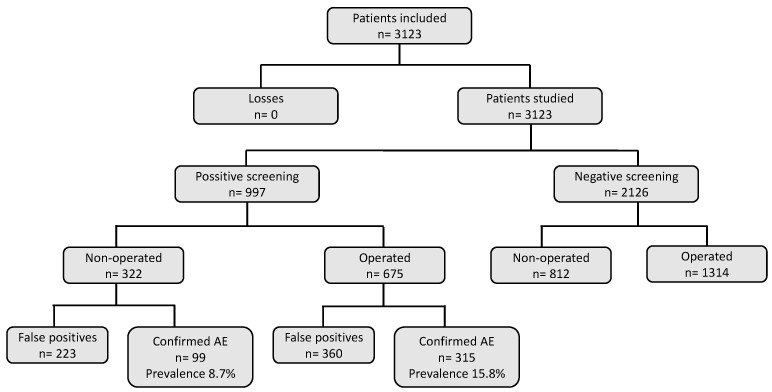
Prevalence of AE in patients seen in surgical services.

Therefore, the prevalence of AE in non-operated patients was 8.7% and the prevalence of AE in operated patients was 15.8%, with statistical significance. (Figure 1).

The total number of AEs detected in the surgical services was 517 (113 AEs in non-operated patients and 404 in operated patients), as some patients referred more than one AE.

The prevalence of AEs in medical services was 11.4%, lower than the prevalence of total AEs in surgical services, regardless of treatment received, which was 13.3%, and the difference reached statistical significance. (Table 1).

Of the total figure of 517 AEs recorded in the surgical services, more than half took place in the inpatient ward (37.45%) as well as during a procedure (22%), being much more frequent in both cases in the group of operated patients. On the other hand, at discharge and on admission to the ward were the times with the lowest concentration of AEs (4.1% and 1.9%, respectively), and these were more frequent at discharge in patients who had not undergone surgery.

When analysing the characteristics of the study population stratified into operated and non-operated patients, gender was distributed very similarly in both groups, with women being of slight predominance (56.3% in the non-operated group and 53.5% in the operated group). Likewise, comorbidity was also very similar in both groups, with comorbid patients being much more numerous (70.1% and 71.9%). In terms of age, operated patients were slightly older than non-operated patients (median age 64 vs. 50 years, with statistical significance). The presence of both intrinsic and extrinsic risk factors was also higher in operated patients.

The distribution by complexity of operated and non-operated patients in the hospitals was very similar. On the other hand, the type of urgent care or planned admission did differ. In the case of non-operated patients, 83.1% were treated as emergencies, whereas in the group of operated patients, the percentage dropped to 42.5%. The average hospital stay was 5.1 days shorter in non-operated patients (7.6 vs. 12.7 days). (Table 2).

When stratifying the operated and non-operated patients according to whether they suffered an AE or not, the gender distribution showed that both male and female operated patients had more AE with a *p* ≤ 0.01. In addition, the indication for surgical treatment was slightly higher in men (65.1% vs. 62.5%) as was the frequency of AE, which was also higher in men (15% vs. 11.6; *p* = 0.003). In non-operated patients with AE, the median age was higher (62 vs. 49 years), whereas in operated patients, the median age was very similar whether or not they had AE (67 vs. 64), with statistical significance in both cases. The distribution by hospital complexity showed that operated patients with AE were twice as many as non-operated patients with AE, with *p* ≤ 0.001. Furthermore, the proportion of AEs was higher in secondary than in tertiary hospitals, both in the operated and non-operated groups (*p* < 0.001 and *p* = 0.213, respectively).

Regarding the type of care, regardless of whether patients were admitted as emergencies or scheduled, AEs were more frequent in operated patients (*p* ≤ 0.001). However, the frequency of AE was higher in emergency surgery (20.6% vs. 12.4%; *p* < 0.001).

On the other hand, comorbidity was similar in all strata, and the percentage of patients with AE was higher among patients with intrinsic risk factors in both the non-operated and operated groups (12.4% and 18%, respectively). The same was true for extrinsic risk factors (11.5% of patients with AE and extrinsic risk factors in the non-operated group versus 3.9% of patients without these risk factors, and 16.8% of patients with AE and extrinsic risk factors in the operated group versus 10.5% of patients without these risk factors).

Finally, the average length of hospital stay was significantly longer in patients operated with AE, 28.7 days compared to 13 days in patients operated without AE, whereas non-operated patients with AE had an average length of stay of 9.7 days compared to 7.1 days in patients without AE (Table 3).

The most frequent AEs in surgical services were those related to hospital-acquired infection (42.63%), followed by those related to a procedure (37.72%), those related to care (14.73%), those related to medication (4.52%), and others (2.16%). In all cases, AEs were more frequent in operated patients. Thus, the frequency of hospital-acquired infection in operated patients was 33.60% compared to 9.04% in non-operated patients, with surgical wound infection being the most frequent. Of the AEs related to a procedure, the most frequent were those classified as other complications after surgery (11% in operated patients and 4.13% in non-operated patients), followed by haemorrhages or haematoma, which, in the operated patients, accounted for 8.05% of the total compared to 0.98% in the non-operated group. The frequency of medication-related AEs in operated patients was 2.95% compared to 1.57% frequency in non-operated patients.

When analysing the preventability, of the total number of patients with AE (15.8% in operated patients and 8.7% in non-operated patients), more than three-quarters, 77.8%, were considered preventable in patients undergoing surgical treatment, whereas less than a quarter, 22.2%, were classified as preventable in patients undergoing conservative treatment, despite not reaching statistical significance.

When analysing the burden of disease associated with AEs, it was found that 48.3% of AEs led to an increase in hospital stay, and of this percentage, surgery was involved in 92.4% of cases, with a *p* ≤ 0.001. However, 34.21% of the non-operated patients had to be readmitted due to AE, in comparison to 9.38% of the operated patients, with a *p* value ≤ 0.001.

In both groups, the most frequent AEs were classified as moderate (43.07% in operated and 48.21% in non-operated), whereas in operated patients, they were followed in frequency by severe AEs (34.76%) and in non-operated patients by mild AEs (32.14%). (Table 4).

A univariate analysis showed that the surgical intervention factor increased the risk of developing an AE. In fact, in the multivariate analysis, being operated on represented 2.3 times the risk of developing an AE.

On the other hand, univariate analysis also showed an increased risk of AE for each additional day of hospital stay, although this could no longer be demonstrated in the multivariate analysis. The same was true for admission to a surgical speciality or female gender, whose risk of AE was higher, but did not reach statistical significance in the multivariate analysis. Admission to a secondary hospital was associated with a 1.5-fold increased risk of AE, which was maintained in the multivariate analysis. Finally, both intrinsic and extrinsic risk factors increased the risk of AE, such that, as the number of factors increased, the risk increased from twice as much with one factor to almost three times as much with three or more factors. (Table 5).

## 4. Discussion

In this study, the estimated overall prevalence of AE in all admitted patients, regardless of the service or treatment received, was 12.1%, whereas in medical services it was 11.4%, and in surgical services, 13.3%, somewhat higher. However, when we analysed the prevalence of patients who underwent surgery in the surgical services, it was 15.8%, whereas in those who did not undergo surgery, it was 8.7%, showing the difference in risk posed by surgical intervention compared to other treatments, confirming the proposed hypothesis.

In 2016, there was an excess of 15.6 million deaths in low- and middle-income countries, of which 5 million were attributed to receiving poor-quality health care [19]. A significant proportion of these deaths were related to surgical treatment.

In Spain, the 2005 ENEAS study found an overall incidence of AE of 9.3%, and the incidence in general surgery services was 10.3%. In these services, 14.8% of patients with an intrinsic risk factor such as comorbidities suffered AE, compared to 7.2% of those with no risk factor. On the other hand, it was observed that the relationship of extrinsic risk factors, which are very frequent in surgery, had a dose–response relationship with the appearance of AE, such that subjects without risk factors presented AE in 7.0%, which increased to 9.9% when there was one factor, to 16.1% when there were two, and to 29.0% when there were three or more factors [20].

Surgery increases risk; the assertion has biological plausibility, as it intensifies the instrumentalisation of clinical practice, as well as opening a door to health care-associated infection.

An in-depth analysis of these AEs shows that most of them occurred in the hospital ward and during a procedure (37.45% and 22%, respectively), with the main group of AEs being present in operated patients. This is logical and to be expected, since the main risk of surgical treatment occurs in the peri-operative period and during care, as already established by Anderson et al. in a systematic review published in 2013 [21].

It was also observed that intrinsic and extrinsic risk factors were more numerous in operated patients. Therefore, the risk of AE in operated patients is increased by the surgical treatment received, the peri-operative care and the risk factors suffered by the patient, as shown in Table 5. In fact, the percentage of patients with AE and intrinsic risk factors was higher in the operated group than in the non-operated group (18% vs. 12.4%), as well as the percentage of patients with AE and extrinsic risk factors, which was also higher in the operated patients (16.8% vs. 11.5%), i.e., operated patients require a more instrumentalised clinical practice and therefore representgreater risk, coinciding with what has been published in the literature [21,22].

This study also shows the so-called dose–response effect, which becomes evident in the multivariate analysis, whereas both intrinsic and extrinsic risk factors increase, the risk increases almost exponentially, with statistical significance. This complicates the usual surgical scenario in which, in addition to aggressive treatment by definition, patients with an increased risk of AE associated with risk factors and comorbidities may be encountered. In surgery, knowing when not to operate is as important as knowing how to operate, and experience in the former is more difficult to acquire [23]. Avoiding doing what is of no value to the patient means reducing extrinsic risk factors, reducing the cost of care, and increasing patient safety [24,25,26].

With regard to the specific type of AE, it was observed that those corresponding to healthcare-related infection (42.63%), closely followed by those related to a procedure (37.72%), were the most frequent in surgical services, such that hospital-acquired infection in operated patients was 33.60% compared to 9.04% in non-operated patients. This is in line with results from similar published studies, such as the Australian study by Kable et al. [27] or the Portuguese study by Sousa p et al. [28].

Given that the prevalence of AE was higher in operated patients, it is not surprising that the analysis shows an increased hospital stay for these patients with an average stay of 28.7 days compared to 13 days for the operated patients without AE.

When analysing the burden of disease associated with AEs, almost half of the AEs (48.3%) resulted in a prolonged length of stay, of which the vast majority (92.4%) were patients who underwent surgery. This important difference was statistically significant and indicates the overburdened work and cost caused by AEs in operated patients.

On the other hand, it should be noted that 34.21% of non-operated patients had to be readmitted due to AE, whereas only 9.38% of operated patients were readmitted, which suggests that surgery, despite being associated with more AE, enables their resolution during the same admission.

In fact, although in both groups, the severity of AE was more frequently classified, almost half, moderately speaking, the group of severe AEs was almost twice as high in those who underwent surgery (34.76% compared to 19.64% in those who did not undergo surgery, with *p* ≤ 0.005).

This reconfirms the hypothesis of this study, with the operation itself being a risk factor for developing AE, but additionally, these AEs are also more serious, causing prolonged hospital stays that could be diagnosed and resolved during the same hospital stay. Indeed, this statement is again confirmed in both the univariate and multivariate analysis, where the surgical intervention presented 2.3 times the risk of developing an AE with a *p*-value ≤ 0.001.

With regard to the type of care received, and as expected, AEs were more frequent in emergency admissions, both in operated patients (20.6% vs. 12.4%) and in non-operated patients (9.9% vs. 3.1%), data consistent with the study by Bellomo et al. [29], which analysed serious AEs in patients undergoing emergency surgery compared to elective surgery, the study by Sjo et al. [30], which looked exclusively at colon cancer treatment or the study by Ozkan et al. [31], which focused on a study of patients over 65 years of age who all underwent emergency vs. elective surgery, among other studies, all of which showed a dramatic increase in complications when surgery was performed under an emergency situation. On the other hand, there are multiple published studies in which fatigue or long working hours may contribute to the increased risk of AE in emergency care [32,33,34], another important factor to consider that adds to the risk. These facts are what condition the recommendation of the American Society of Surgery, which proposes to reduce urgent interventions to a minimum, carrying out only those that are truly indicated (when not intervening at that moment would pose a risk to the patient’s health or condition the prognosis of the disease) [35].

When analysing the care received by hospital complexity, the proportion of AEs was higher in secondary hospitals than in tertiary hospitals, probably because complex pathologies are not operated on in primary hospitals but in secondary and tertiary hospitals, while secondary hospitals do not have the same availability of health care resources as tertiary hospitals.

Finally, when analysing preventability, more than three quarters of AEs, 77.8%, were considered preventable in patients undergoing surgical treatment, whereas less than a quarter, 22.2%, were classified as preventable in patients undergoing conservative treatment.

The lack of statistical significance in this case could suggest that AEs in surgery or associated with surgery are inevitable and inherent to the risk involved in the intervention itself; however, there is always ample room for progress and study to obtain proposals for improvement that subsequently show good results. Proof of these are the many programmes that are widely extended due to their proven effectiveness, such as the worldwide application of the checklist [36,37,38], the zero surgical infection in Spain [39], the SURPASS protocol (SURgical PAtient Safety System) in Sweden [40,41], the marking of the surgical site, if possible, with the patient’s own collaboration [42], the visualisation of CT, X-ray or other images during the intervention [43], or patient education programmes at discharge on stomas and drainage systems to reduce unnecessary consultations and readmissions [44], among others.

Surgical treatment, in addition to having an enormous weight in health care, presents a series of peculiarities that have a significant influence on patient safety. On the one hand, published studies point to surgical specialities as the cause of most AEs, which is justified by the magnitude of the aggression that a surgical intervention represents in itself, but it should not be forgotten that it is the treatment in which the greatest number of professionals participate and intervene with the need to coordinate among each other in a short period of time, as well as in critical moments and at times of great stress. Added to this is the great complexity of many of the pathologies presented in increasingly older and comorbid patients, and therefore with a greater number of intrinsic and extrinsic risk factors.

On the other hand, there is a not insignificant component of personal involvement, due to the fact that the treatment is carried out by the main surgeon who is responsible for the evolution of the postoperative scenario, which, in many cases, leads to a feeling of incrimination.

Therefore, it is important that health policies focus on surgical areas, not in a punitive sense, but in the sense of supporting the professionals who work and the patients who are cared for in this area in order to continue with the development of plans and protocols aimed at improving the quality of care and patient safety [45].

This study is pioneering in analysing the effect of surgical interventions on the development of AE. This had not been done in any previous study using the standardized, replicated and validated methodology designed by Brennan et al.

The limitations of this study derive mainly from its cross-sectional design [46], which, despite being more efficient in terms of time and resources and easier to carry out, does not allow for the study of the entire hospitalisation episode, which makes it likely to underestimate shorter and/or milder AEs and overestimate severe or long resolution periods. Despite the above, this design has proven to be able to maintain a more stable observing system over time. In addition, communication with health care staff makes it easier to judge the causality of AE and its preventability, as the patient is hospitalised at the time of the study.

On the other hand, the fact that the prevalence design detects proportionally more severe AEs is not a drawback, as these are precisely the AEs that should be prioritised when designing control strategies.

## 5. Conclusions

To conclude, although surgical sites are particularly vulnerable to AE, the role of surgical treatment in this area has never been analysed. Surgical intervention alone is a risk factor for AE, and we must continue to work to improve the safety of both patient care and the working environment of surgical professionals.

## Figures and Tables

**Table 1:** Prevalence of AE in medical and surgical services.

Speciality	AE		
	No AE	AE	Total
Medical	4593 (88.6%)	591 (11.4%)	5184 (100%)
Surgical	2709 (86.7%)	414 (13.3%)	3123 (100%)
Total	7302 (87.9%)	1005 (12.1%)	8307 (100%)

**Table 2:** Characteristics of the study population in operated and non-operated patients.

		Operation			
		No (%)	Yes (%)	Total	*p*
Gender	Female	638 (56.3%)	1064 (53.5%)	1702 (54.5%)	*p* = 0.135
	Male	496 (43.7%)	925 (46.5%)	1421 (45.5%)	
	Total	1134 (100%)	1989 (100%)	3123 (100%)	
Age	Mean (SD)	48.5 (28)	60.7 (20.2)	56.2 (24.1)	*p* < 0.001
	Median (IR)	50 (30–73)	64 (48–76)	61 (40–75)	
Hospital Complexity	Tertiary	682 (60.1%)	1193 (60%)	1875 (60%)	*p* = 0.111
	Secondary	345 (30.4%)	634 (31.9%)	979 (31.4%)	
	Primary	88 (7.8%)	116 (5.8%)	204 (6.5%)	
	Support	19 (1.7%)	46 (2.3%)	65 (2.1%)	
	Total	1134 (100%)	1989 (100%)	3123 (100%)	
Admission type	Unplanned admission	936 (83.1%)	843 (42.5%)	1779 (57.2%)	*p* < 0.001
	Planned admission	191 (16.9%)	1139 (57.5%)	1330 (42.8%)	
	Total	1127 (100%)	1982 (100%)	3109 (100%)	
Number of intrinsic risk factors	0	458 (40.4%)	424 (21.3%)	882 (28.2%)	*p* < 0.001
	1	206 (18.2%)	470 (23.7%)	676 (21.6%)	
	2	162 (14.3%)	416 (21%)	578 (18.5%)	
	3 or more	308 (27.2%)	679 (34.1%)	987 (31.6%)	
	Total	1134 (100%)	1989 (100%)	3123 (100%)	
Number of extrinsic risk factors	0	412 (36.3%)	314 (15.8%)	726 (23.2%)	*p* < 0.001
	1	591 (52.1%)	1109 (55.8%)	1700 (54.4%)	
	2	126 (11.1%)	436 (22%)	562 (18%)	
	3 or more	5 (0.5%)	130 (6.5%)	135 (4.3%)	
	Total	1134 (100%)	1989 (100%)	3123 (100%)	
Patient comorbidity	No	85 (29.9%)	155 (28.1%)	240 (28.7%)	*p* = 0.608
	Yes	200 (70.1%)	396 (71.9%)	596 (71.3%)	
	Total	285 (100%)	551 (100%)	836(100%)	
Number of patient comorbidity	0	85 (29.9%)	155 (28.1%)	240 (28.7%)	*p* = 0.343
	1	52 (18.2%)	78 (14.2%)	130 (15.6%)	
	2	39 (13.7%)	84 (15.2%)	123 (14.7%)	
	3 or more	109 (38.2%)	234 (42.5%)	343 (41%)	
	Total	285 (100%)	551 (100%)	836 (100%)	
Length of stay until the day of study	Mean (SD)	7.6 (34.7)	12.7 (34.3)	10.9 (34.5)	*p* < 0.001
	Median (IR)	3 (1–7)	4 (2–12)	4 (1–10)	

**Table 3:** Characteristics of the study population of operated and non-operated patients with and without AE.

		Operation								
		No				Yes				
		Patients without AE		Patients with AE		Patients without AE		Patients with AE		Chi-Square *p*-Value
		*n*	%	*n*	%	*n*	%	*n*	%	
Gender	Female	587	92	51	8	917	86.2	147	13.8	*p* < 0.001
	Male	448	90.3	48	9.7	757	81.8	168	18.2	*p* < 0.001
Age	Mean (SD)	47.5	28.5	58.2	20.25	60.1	20.3	63.8	19	
	Median (IR)	49	29.73	62	42.74	64	48.76	67	55.78	
	U Mann–Whitney	*p* = 0.001				*p* = 0.0025				
Hospital Complexity	Tertiary	628	92.1	54	7.9	1027	86.1	166	13.9	*p* < 0.001
	Secondary	308	89.3	37	10.7	502	79.2	132	20.8	*p* < 0.001
	Primary	83	94.3	5	5.7	101	87.1	15	12.9	*p* = 0.085
	Support	16	84.2	3	15.8	44	95.6	2	4.3	*p* = 0.115
Admission type	Unplanned admission	843	90.1	93	9.9	669	79.4	174	20.6	*p* ≤ 0.001
	Planned admission	185	96.9	6	3.1	998	87.6	141	12.4	*p* < 0.001
Intrinsic risk factors	No	443	96.7	15	3.3	390	92	34	8	*p* = 0.002
	Yes	592	87.6	84	12.4	1284	82	281	18	*p* < 0.001
Number of intrinsic risk factors	0	443	96.7	15	3.3	390	92	34	8	*p* = 0.002
	1	184	89.3	22	10.7	411	87.5	59	12.5	*p* = 0.490
	2	135	83.3	27	16.7	356	85.6	60	14.4	*p* = 0.498
	3 or more	273	88.6	35	11.4	517	76.1	162	23.9	*p* < 0.001
Extrinsic risk factors	No	396	96.1	16	3.9	281	89.5	33	10.5	*p* < 0.001
	Yes	639	88.5	83	11.5	1393	83.2	282	16.8	*p* < 0.001
Number of extrinsic risk factors	0	396	96.1	16	3.9	281	89.5	33	10.5	*p* < 0.001
	1	530	89.7	61	10.3	940	84.8	169	15.2	*p* = 0.005
	2	104	82.5	22	17.5	350	80.3	86	19.7	*p* = 0.570
	3 or more	5	100	0	0	103	79.2	27	20.8	*p* = 0.255
Patient comorbidity	No	64	75.3	21	24.7	105	67.7	50	32.2	*p* = 0.220
	Yes	151	75.5	49	24.5	257	64.9	139	35.1	*p* = 0.009
Number of patient comorbidity	0	64	75.3	21	24.7	105	67.7	50	32.2	*p* = 0.220
	1	43	82.7	9	17.3	46	59	32	41	*p* = 0.004
	2	25	64.1	14	35.9	57	67.9	27	32.1	*p* = 0.681
	3 or more	83	76.2	26	23.8	154	65.8	80	34.2	*p* = 0.054
Length of stay until the day of study	Mean (SD)	7.1	35.7	13	21.7	9.7	33.5	28.7	34.2	
	Median (IR)	2	1.6	6	3.14	3	1.8	17	8.36	
	U Mann–Whitney	*p* ≤ 0.001				*p* ≤ 0.001				

**Table 4:** Impact of AEs.

		Operation			
		No (%)	Yes (%)	Total	*p*
Prolonged hospital stay	No	94 (35.1%)	174 (64.9%)	268	*p* ≤ 0.001
	Yes	19 (7.6%)	231 (92.4%)	250	
Extra days same hospitalisation	Mean (SD)	9.63 (18.6)	15.78 (29.7)	14.45 (27.8)	0.178
	Median (IR)	6 (0.13)	5 (0.20)	6 (0.17)	
Causing admission	No	50 (65.79%)	309 (90.61%)	359	*p* ≤ 0.001
	Yes	26 (34.21%)	32 (9.38%)	58	
Extra days new hospitalisation	Mean (SD)	11.7 (5.84)	27.2 (35.7)	20.2 (27.7)	0.368
	Median (IR)	11 (7.15)	15 (3.30)	12 (6.20)	
Severity	Mild	36 (32.14%)	88 (22.16%)	124	0.005
	Moderate	54 (48.21%)	171 (43.07%)	225	
	Severe	22 (19.64%)	138 (34.76%)	160	
Preventable	No	32 (24.1%)	101 (75.9%)	133	0.686
	Yes	45 (22.2%)	158 (77.8%)	203	

**Table 5:** Univariate and multivariate logistic regression.

	Univariate			Multivariate (N = 7836)		
Variables	OR	95% CI for OR	*p*-Value	OR	95% CI for OR	*p*-Value
Operation	1.77	1.54–2.02	*p* ≤ 0.001	2.30	1.88–2.83	*p* ≤ 0.001
Length of stay until the day of study	1.00	0.99–1.00	*p* ≤ 0.001	1.00	0.99–1.00	0.857
Department (reference: medical speciality)	1.18	1.04–1.36	0.012	0.95	0.78–1.16	0.605
Gender (reference: female)	1.15	1.01–1.32	0.031	1.05	0.92–1.21	0.472
Hospital complexity (primary hospital reference)						
Secondary	1.54	1.16–2.03	0.002	1.49	1.12–1.98	0.006
Tertiary	1.07	0.82–1.41	0.613	1.02	0.77–1.35	0.887
Type of admission (reference, planned)	1.15	0.98–1.34	0.074			
Number of intrinsic risk factor (none)						
1	2.26	1.69–3.01	*p* ≤ 0.001	1.98	1.47–2.67	*p* ≤ 0.001
2	2.49	1.88–3.30	*p* ≤ 0.001	2.18	1.63–2.93	*p* ≤ 0.001
≥3	3.21	2.51–4.10	*p* ≤ 0.001	2.97	2.28–3.86	*p* ≤ 0.001
Number of extrinsic risk factor (none)						
1	1.93	1.54–2.42	*p* ≤ 0.001	1.42	1.11–1.81	0.005
2	3.72	2.91–4.77	*p* ≤ 0.001	2.37	1.82–3.09	*p* ≤ 0.001
≥3	4.58	3.17–6.61	*p* ≤ 0.001	2.58	1.74–3.83	*p* ≤ 0.001

## Data Availability

The database can be made available upon request to the principal investigator.
